# INCIDENCE OF HERPES SIMPLEX VIRUS KERATITIS AND OTHER OCULAR DISEASE: GLOBAL REVIEW AND ESTIMATES

**DOI:** 10.1080/09286586.2021.1962919

**Published:** 2021-10-08

**Authors:** Ian McCormick, Charlotte James, Nicky J Welton, Philippe Mayaud, Katherine M. E Turner, Sami L Gottlieb, Allen Foster, Katharine J Looker

**Affiliations:** a Department of Clinical Research, Faculty of Infectious and Tropical Diseases, London School of Hygiene & Tropical Medicine, London, UK; b Population Health Sciences, Bristol Medical School, University of Bristol, Bristol, UK; c Bristol Veterinary School, University of Bristol, Bristol, UK; d Department of Sexual and Reproductive Health And Research, World Health Organization, Geneva, Switzerland

**Keywords:** Herpes simplex virus, ocular HSV, keratitis, incidence, burden

## Abstract

Purpose: We aimed to review available data on the incidence of herpes simplex virus (HSV) keratitis and other HSV ocular disease and to estimate the global burden of HSV ocular disease. Methods: We searched Medline and Embase databases to October 2020 for studies reporting on the incidence of HSV ocular disease. Study quality was evaluated using a four-point checklist. Pooled estimates were applied to 2016 population data to estimate global HSV ocular disease burden. Numbers with uniocular vision impairment (any visual acuity <6/12) were estimated by applying published risks to case numbers. Results: Fourteen studies had incidence data; seven met our quality criteria. In 2016, an estimated 1.7 (95% confidence interval, 95% CI 1.0–3.0) million people had HSV keratitis, based on a pooled incidence of 24.0 (95% CI 14.0–41.0; N = 2; I^2^ = 97.7%) per 100,000 person-years. The majority had epithelial keratitis (pooled incidence 16.1 per 100,000; 95% CI 11.6–22.3; N = 3; I^2^ = 92.6%). Available studies were few and limited to the USA and Europe. Data were even more limited for HSV uveitis and retinitis, although these conditions may collectively contribute a further >0.1 million cases. Based on global incidence, some 230,000 people may have newly acquired uniocular vision impairment associated with HSV keratitis in 2016. Conclusion: Over 1.8 million people may have herpetic eye disease annually. Preventing HSV infection could therefore have an important impact on eye health. Herpetic eye disease burden is likely to have been underestimated, as many settings outside of the USA and Europe have higher HSV-1 prevalence and poorer access to treatment.

## Introduction

Herpes simplex virus (HSV) exists as two types – type 1 (HSV-1) and type 2 (HSV-2). Worldwide, there are an estimated 4.85 billion people of all ages with prevalent HSV-1 infection.^1^ Although endemic across the globe, prevalence varies by region, being highest in the World Health Organization (WHO) Africa region and lowest in the WHO Americas region.^1^ There are an estimated 836 million people aged 15 years and above with prevalent HSV-2 infection, with prevalence again highest in the WHO Africa region.^1^ Infection with HSV is life-long, and is characterised by periodic recurrences of disease in a proportion of those infected.^2,3^

HSV-1 is predominately transmitted by the oral-oral route, establishes latency in the trigeminal ganglion, and is associated with orolabial, ocular, and neurological conditions.^4^ HSV-1 is also an increasingly frequent cause of genital infection, through oral sex.^5,6^ HSV-2 is almost exclusively a sexually transmitted infection which establishes latency in the sacral ganglia and is associated with genital ulcer disease, but can occasionally cause extra-genital infection.^2^

Ocular disease can result from a primary HSV infection of the eye or accompany a primary extra-ocular HSV infection. However, it typically occurs following reactivation of latent infection acquired at an extra-ocular oral or facial site.^7^ HSV-1 is a much more common cause of ocular disease than HSV-2.^8^ Diagnostic testing for HSV can be done by direct identification from lesions using viral culture or nucleic acid amplification tests (NAAT) such as polymerase chain reaction (PCR), or serologically using type-specific enzyme-linked immunosorbent (ELISA) or immunofluorescence antibody (IFA) assays. Molecular testing by PCR has been shown to improve diagnostic sensitivity over clinical examination alone, however, identification of the virus, or virus typing, is seldom undertaken in a clinical setting, with ophthalmologists relying on pathognomonic clinical signs as the means of diagnosing HSV ocular disease.^9^

HSV can affect different ocular structures resulting in clinical entities of varying severity.^10^ The principal ocular complication of HSV is keratitis (infection of the cornea), which takes three forms: epithelial, stromal, or, more rarely, endothelial. Epithelial (dendritic or geographic) keratitis affects the superficial cornea and, although recurrences are common, vision usually remains unaffected if only the epithelium is involved (around 9% have mild or greater vision impairment, defined as any visual acuity (VA) <6/12.^11^) An immune response to HSV infection can affect the deeper stromal layer of the cornea (with or without ulceration) and the resulting inflammation is more difficult to treat. Stromal keratitis is more likely to result in vision impairment (around 24–42% <6/12.^11–14^) Endothelial (or disciform) keratitis is caused by an immune response that affects the endothelial cells resulting in oedema of the cornea. Farooq et al., in a 2012 review, estimated that 1.5%–3.0% of HSV keratitis cases (considering all possible forms of keratitis) lead to severe vision impairment (defined as VA <6/60) in the affected eye, based on tertiary-level data.^8^

Herpes viruses, including HSV-1 and HSV-2, can also occasionally cause anterior uveitis (iritis or iridocyclitis) and more rarely retinitis, in the form of acute retinal necrosis (ARN) or progressive outer retinal necrosis (PORN), although both uveitis and retinitis have multiple other aetiologic agents besides HSV.^10^ ARN and PORN can lead to retinal detachment and significant visual morbidity in the affected eye.^15–17^ HSV blepharo-conjunctivitis is self-limiting with low morbidity and can present as a vesicular rash on the eyelids, which may be associated with a follicular conjunctivitis.^10^ Blepharo-conjunctivitis in early life is the typical clinical manifestation of a primary ocular HSV infection.^18^

Efforts to quantify the global and regional burden of HSV-related disease are being undertaken to better understand the disease burden that could potentially be prevented or ameliorated with the development of effective HSV interventions such as vaccines.^19^ HSV ocular disease represents an important component of HSV-related morbidity. The Farooq review in 2012 estimated the annual number of cases of herpetic keratitis globally to be 1.5 million, based on extrapolating data from two studies in high income settings to global population numbers.^8^ Our aim was to update and expand upon that review to summarise available data on the incidence of new and/or recurrent HSV keratitis, as well as other HSV ocular disease, and to estimate the global burden of HSV ocular disease.

## Methods

### Eligibility criteria for considering studies for this review

We aimed to review available data on the incidence of HSV ocular disease through a systematic literature search. We also aimed to summarise the best-available data through a quantitative meta-analysis to estimate the global burden of HSV keratitis and other ocular disease where appropriate. We considered the following clinical entities attributable to HSV (or specifically HSV-1 or HSV-2 where typed): keratitis (evaluating the different forms separately where possible), uveitis, retinitis, and blepharo-conjunctivitis. We considered both new disease (defined as the first episode) and recurrent disease (defined as a subsequent episode). We considered data for all ages >1 month old, and all geographic locations and study years. Neonatal HSV studies were excluded, as the burden of neonatal herpes has been separately quantified.^20^

### Search methods for identifying studies

Search terms for HSV ocular disease are listed in the Supplemental online material. The search strategy was refined through a scoping search and co-author review and was focused on HSV-specific ocular disease; we did not attempt to search for all-cause disease incidence and, separately, the fraction attributable to HSV. No restrictions were placed at the search stage to eliminate neonatal studies; instead such studies were identified during abstract or full text screen. We searched Medline and Embase databases from 1946 and 1947 respectively up to 23^rd^ October 2020 for relevant published studies in the English language. Conference abstracts were excluded. Reference lists of key papers were also searched. Endnote X9 was used to manage the publications identified and their eligibility status.

### Study selection

Studies were included in the review if they reported data on disease incidence for any clinical entity of interest. Only studies reporting incidence based on the number of cases for a specified, general population and defined time period were included. Data were not extracted for studies only including people living with HIV, on the basis that these individuals may not be representative of the general population. Abstract screening and full text screening were performed by one researcher (IM).

### Data collection and risk of bias assessment

We extracted data on study year(s), location and population, sample size, study design, study population age, study strengths and limitations, number of (HSV) cases of disease, and estimate(s) of incidence and confidence measure around that estimate (95% confidence interval, 95% CI). Data were extracted both for individual clinical entities, and for HSV ocular disease not separated out by clinical entity or form (e.g., ‘any HSV ocular disease’ or ‘any keratitis’). For each clinical entity, incidence data were extracted for new cases, recurrent cases, and ‘all’ cases, as far as possible. As confirmation of HSV type was not expected to be routinely available, we extracted data available for any HSV infection, but additionally separated findings by HSV-1 and HSV-2 where possible. Data from included studies were extracted by one researcher (IM) and checked by a second (CJ).

To evaluate each study for inclusion in our set of best-available estimates and potential pooled estimates, we designed and conducted a risk of bias (quality) assessment, using the Newcastle-Ottawa scale as a guide.^21^ This assessment evaluated whether (1) cases were well defined, (2) the study population (denominator) was well defined, (3) the study was designed to capture all cases within the study population, (4) the cases and population represented a general population, without major selection, referral, or design bias (Supplemental online material). Studies had to meet all four criteria in order to potentially be included in our pooled estimates. The risk of bias assessment was done by one researcher (IM).

### Data synthesis and analysis

We first described the incidence of HSV keratitis and other ocular disease as reported by individual studies, in the context of study strengths and limitations. Next, for each clinical entity where at least two separate study estimates were available, we used meta-analysis to pool those that met certain methodological standards (‘best-available estimates’). We did not limit the pooling based on year of study, given the limited number of recent studies and several studies spanning many years to capture infrequent outcomes. We then applied the pooled best-available estimates to 2016 global population data to provide estimates of global HSV ocular disease burden.

Analysis was done using Stata 15 software. Incidence was pooled on the log scale, with the standard error (SE) of the log incidence calculated according to the formula 1/sqrt(cases). Pooled estimates were exponentiated to obtain incidence on the natural scale. Pooling was done using the metaan command, assuming a random-effects model. Heterogeneity between studies was explored with the I^2^ statistic. This is the percentage of the variation in the estimates explained by between-study variation. Cochrane’s Q test was used to assess whether the estimates were statistically significantly different between studies.

### Estimation of global burden of HSV ocular disease

Pooled best-available estimates of incidence were used to estimate the number of people aged 1 month to 99 years with HSV ocular disease in 2016, by applying the estimates to global population numbers for 2016 obtained from the United Nations Population Division.^22^ 95% CI were calculated for each burden estimate based on the 95% CI for the pooled estimates. The year 2016 was chosen to align with the most recent HSV-1 and HSV-2 infection estimates,^1^ and other HSV disease burden estimates.^23^ We also estimated the number of people with at least mild uniocular vision impairment attributable to epithelial and stromal HSV keratitis, using percentages from studies reporting vision impairment <6/12 identified through our literature search (Supplemental online material). Two studies reported the proportion of eyes affected by epithelial keratitis. One study reported an estimate of 8.9%^11^; we did not use the other estimate which was derived from a sample of young children only.^24^ In three studies reporting vision impairment <6/12 from stromal HSV keratitis,^11,13,14^ the proportion of eyes affected ranged from 28%-42%; in a fourth study it was unclear how much vision impairment was attributable to stromal HSV keratitis.^12^ These three estimates were pooled to give an overall estimate of 35.7% (95% CI 29.9–42.0) for vision impairment <6/12 due to stromal HSV keratitis. We assumed that, as the majority of keratitis cases are unilateral, the percentage of eyes with vision impairment following HSV keratitis was similar to the percentage of individuals with uniocular vision impairment.

Our estimates followed the Guidelines for Accurate and Transparent Health Estimates Reporting (GATHER)^25^ and the Meta-analysis Of Observational Studies in Epidemiology (MOOSE) checklist^26^ and were reported with reference to the PRISMA^27^ checklist.

## Results

### Literature search

**Figure 1.**
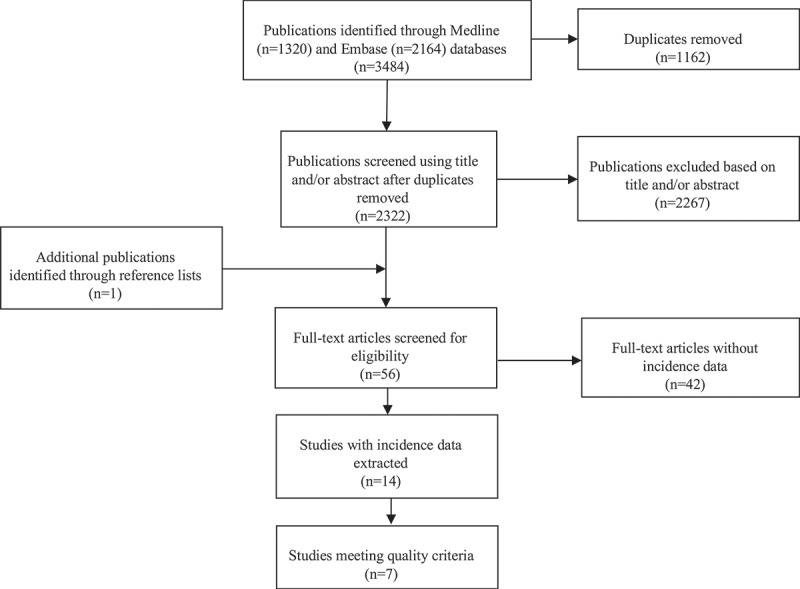
Flow diagram of HSV ocular disease literature search with number of studies at each stage of the process.

A total of 3484 publications were identified in the literature search (Figure 1). After 1162 duplicates were removed, the remaining 2322 publications were screened using title and/or abstract. Of these, the full text was retrieved for 55 studies. One of these referenced a further publication that was also retrieved. Of these 56 publications, 14 publications (14 studies) were able to contribute data on incidence.^11,15,16,28–38^ Seven of these studies met our quality criteria for best-available estimates.^15,16,28,30,32,35,36^ Incidence data from all 14 available studies are shown in Supplemental online material; Table 1 summarises the estimates from the 7 studies meeting our quality criteria. The quality assessment is detailed in Supplemental online material. Data on uniocular vision impairment <6/12 extracted from studies identified in the search are shown in Supplemental online material.^11–14,24,28,39–41^

**Table 1.** Number of available estimates from the 7 studies meeting quality criteria, and pooling decision.

HSV clinical entity	All cases	New cases	Recurrent cases	Pooling and estimation of global burden done?
Any ocular disease	2	3	2	No; disease definition not specific
Any keratitis	2^a^	3	2	Yes; for all categories
Epithelial keratitis	3	2	1	Yes for all and new cases, no for recurrent cases due to only one available estimate
Stromal keratitis	2	2	1	Yes for all and new cases, no for recurrent cases due to only one available estimate
Endothelial keratitis	0	0	0	No; no available estimates
Uveitis	1	2	1	Yes for new cases, no for all and recurrent cases due to only one available estimate
Retinitis	0	3	0	Yes for new cases, no for all and recurrent cases due to no available estimates
Blepharo-conjunctivitis	1	2	1	Yes for new cases, no for all and recurrent cases due to only one available estimate

^a^Additional estimate based on highly probable (only) cases also available but not included in tally.

### HSV ocular disease incidence estimates

The 14 studies of HSV ocular disease incidence dated from the 1950s up to the 2010s (Supplemental online material).^11,15,16,28–38^ Most were conducted in Europe or the USA. Studies focussed mainly on HSV keratitis, or HSV ocular disease without differentiation by clinical entity, although there were two studies each reporting on retinitis or uveitis only. Altogether, 46 individual incidence estimates were reported or could be calculated from the 14 studies. When considering only those seven studies that met our quality criteria (Supplemental online material),^15,16,28,30,32,35,36^ the number of available estimates was 36 (this excluded one incidence estimate based on highly probable cases from an included paper with other included estimates.^36^) (Table 1).

Hereafter, we focus only on those “best-available” estimates meeting our quality criteria.

Incidence estimates meeting our quality criteria were available across most HSV ocular clinical entities of interest but were limited in number: seven estimates for any keratitis (2 all,^28,36^ 3 new,^28,32,36^ 2 recurrent^28,36^); six for epithelial keratitis (3 all,^28,30,36^ 2 new,^28,32^ 1 recurrent^28^); five for stromal keratitis (2 all,^28,36^ 2 new,^28,32^ 1 recurrent^28^); four for uveitis (1 all,^28^ 2 new,^28,32^ 1 recurrent^28^); three for retinitis (new^15,16,32^); and four for blepharo-conjunctivitis (1 all,^28^ 2 new,^28,32^ 1 recurrent^28^) (Supplemental online material). We found no estimates for endothelial keratitis. There were seven estimates for any HSV ocular disease: 2 for all,^28,35^ 3 for new^28,32,35^ and 2 for recurrent disease.^28,35^ Estimates for any HSV ocular disease represent unknown proportions of separate clinical entities and as such are less informative in estimating the burden of disease. All the studies meeting our quality criteria were conducted in either Europe or the USA, with no restrictions in any study on participant age (range of available means: 37–54 years). The majority of studies explicitly or seemingly derived incidence based on the number of individuals with disease, i.e., counting individuals no more than once, although some studies specified that their incidence estimates were derived by counting the total number of disease episodes.

#### HSV keratitis

Four studies reported on HSV keratitis incidence: from Europe (2 studies: 1 national,^36^ 1 county-based^30^) and the USA (2 studies in the same county at two different time points,^28,32^) with earliest date of data collection in the 1950s (1 study,^28^) the 1970s (2 studies^30,32^) and the 2000s (1 study^36^) (Supplemental online material). Two studies were retrospective reviews of medical records linkage systems^28,32^; two were prospective multi-centre case series studies.^30,36^

The two available estimates of the incidence of any keratitis (all, i.e., new or recurrent combined) were 18.2 cases per 100,000 person-years.^28^ and 315 cases per 100,000 person-years,^36^ but based on the number of episodes and the number of individuals respectively (Supplemental online material). A further difference is that the former estimate was calculated by combining the separate incidences of epithelial and stromal keratitis, while the latter also included endothelial keratitis (although incidence of “other” keratitis in the study, which included endothelial keratitis, was rare). The corresponding latter study also reported an estimate based on highly probable cases only of 25.8 cases per 100,000 person-years,^36^ which was used by Farooq et al.^8^ We did not use this estimate, however, in favour of that based on all cases, for consistency with the other estimates. The same two studies also reported on (or provided data enabling the calculation of) the incidence of any new keratitis: 6.2 cases per 100,000 person-years.^28^ and 132 cases per 100,000 person-years,^36^ and the calculation of the incidence of any keratitis for recurrent cases: 12.0 cases per 100,000 person-years,^28^ and 183 cases per 100,000 person-years.^36^ In addition, a third study also reported an incidence estimate for any new keratitis of 9.2 per 100,000 person-years.^32^

Three studies reported on the incidence of all epithelial HSV keratitis (Supplemental online material).^28,30,36^ Two of these studies were the same studies as those with estimates of any keratitis (new and recurrent^28,36^): estimates for all epithelial keratitis were 15.6 cases per 100,000 person-years based on the number of episodes^28^ and 220 cases per 100,000 years derived by counting the number of individuals.^36^ Comparing the incidence of any keratitis and epithelial keratitis within each of the two studies indicated that epithelial keratitis contributed the most to keratitis incidence: stromal HSV keratitis incidence in these studies was 2.6^28^ and 9.2 cases per 100,000 person-years respectively.^36^ The other study reporting on the incidence of all epithelial HSV keratitis was a study reporting only on epithelial keratitis and had an estimate of the incidence of 12.0 cases per 100,000 person-years.^30^ Liesegang et al.^28^ also reported separate incidences for new epithelial keratitis and new stromal keratitis, from which we also calculated the incidences of recurrent epithelial and stromal keratitis, while Young et al.,^32^ which reported on the same population as Liesegang et al. but for a later time period, presented data enabling the calculation of the incidence of new epithelial keratitis and new stromal keratitis.

#### Other HSV ocular clinical entities

One study^28^ reported on the incidence of all HSV uveitis (1.9 cases per 100,000 person-years) and all HSV blepharo-conjunctivitis (7.7 cases per 100,000 person-years) based on the number of episodes (Supplemental online material). This study also reported on the incidence of new HSV uveitis (0.4 cases per 100,000 person-years) and new HSV blepharo-conjunctivitis (4.2 cases per 100,000 person-years), while derived incidences from the study which reported on the same population but for a later time period^32^ were 0.06 and 2.4 cases per 100,000 person-years for new HSV uveitis and new HSV blepharo-conjunctivitis respectively. In both studies, HSV uveitis was defined by the presence of prior or concurrent corneal or other ocular HSV involvement.

Two further studies reported (only) on the incidence of new HSV retinitis: these estimates were 0.013 cases per 100,000 person-years^16^ and 0.01–0.012 cases per 100,000 person-years.^15^ Young et al. reported finding no cases of new HSV retinitis.^32^ One other study^35^ reported only on any HSV ocular disease and did not provide any estimates for individual clinical entities.

### The global burden of HSV ocular disease in 2016

Across available estimates, the pooled incidence of any HSV keratitis (new and recurrent combined) was 24.0 cases per 100,000 person-years (95% CI 14.0–41.0; number of contributing studies, N = 2; I^2^ = 97.7%; *p* < .001) (Table 2). Applying this pooled incidence universally across the global population (7.284 billion) gave an estimated 1.7 million cases of any HSV keratitis (95% CI 1.0–3.0) among 1 month to 99 year olds worldwide in 2016.

**Table 2.** Pooled incidence and estimated global burden among 1 month-99 year olds in 2016 for selected clinical entities.

HSV clinical entity	Pooled incidence (95%CI) (cases per 100,000 person-years)	No. of contributing studies	I^2^	Cochrane’s Q test	Estimated no. of global cases among 1 month-99 year olds in 2016 in millions (95%CI)
Any keratitis	24.0 (14.0–41.0)	2^28, 36^	97.7%	p < .001	1.7 (1.0–3.0)
Any new keratitis	9.2 (6.4–13.2)	3^28, 32, 36^	93.6%	p < .001	0.7 (0.5–1.0)
Any recurrent keratitis	14.8 (9.8–22.4)	2^28, 36^	93.8%	p < .001	1.1 (0.7–1.6)
All epithelial keratitis	16.1 (11.6–22.3)	3^28, 30, 36^	92.6%	p < .001	1.2 (0.8–1.6)
New epithelial keratitis	6.4 (5.2–7.9)	2^28, 32^	65.3%	p = .090	0.5 (0.4–0.6)
All stromal keratitis	4.9 (1.4–17.0)	2^28, 36^	97.5%	p < .001	0.4 (0.1–1.2)
New stromal keratitis	1.1 (0.4–3.5)	2^28, 32^	88.1%	p = .004	0.1 (0.0–0.3)
New uveitis	0.2 (0.0–1.1)	2^28, 32^	80.6%	p = .023	0.0 (0.0–0.1)
New retinitis	0.01 (0.01–0.02)	3^15, 16, 32^	0.0%	p = .949	0.00 (0.00–0.00)
New blepharo-conjunctivitis	3.2 (1.8–5.5)	2^28, 32^	91.1%	p = .001	0.2 (0.1–0.4)

Values do not necessarily sum across rows due to different studies contributing estimates, and potential contribution of endothelial keratitis to estimates for any keratitis. I^2^ is the percentage of the variation between the estimates explained by between-study variation (i.e., it is a measure of the heterogeneity between studies). Cochrane’s Q test assesses whether the estimates are significantly different between studies. All values shown to 1 d.p., except for pooled incidence estimates and estimated number of global cases for new retinitis which are shown to 2 d.p.

Any new HSV keratitis had a pooled incidence of 9.2 cases per 100,000 person-years (95% CI 6.4–13.2; N = 3; I^2^ = 93.6%; *p* < .001) or 0.7 million cases (95% CI 0.5–1.0) in 2016, while the pooled incidence of any recurrent HSV keratitis was 14.8 cases per 100,000 person-years (95% CI 9.8–22.4; N = 2; I^2^ = 93.8%; *p* < .001) or 1.1 million cases (95% CI 0.7–1.6) (Table 2).

All epithelial HSV keratitis had a pooled incidence of 16.1 cases per 100,000 person-years (95% CI 11.6–22.3; N = 3; I^2^ = 92.6%; *p* < .001), equivalent to 1.2 million global cases (95% CI 0.8–1.6) in 2016. All stromal HSV keratitis had an incidence of 4.9 cases per 100,000 person-years (95% CI 1.4–17.0; N = 2; I^2^ = 97.5%; *p* < .001) or 0.4 million cases (95% CI 0.1–1.2) (Table 2). Of these, pooled incidence was 6.4 cases per 100,000 person-years for new epithelial HSV keratitis and 1.1 cases per 100,000 person-years for new stromal HSV keratitis.

For the remaining clinical entities, we were only able to produce pooled incidence estimates for new cases (Table 2). However, the one available estimate of incidence for all uveitis (1.9 per 100,000 person-years), from Liesegang et al.,^28^ would translate into approximately 0.1 million cases in 2016 (Supplemental online material). The one available estimate of incidence for all blepharo-conjunctivitis (7.7 per 100,000 person-years), also from Liesegang et al.,^28^ would translate into approximately 0.6 million cases in 2016. HSV retinitis seems to be rare, based on the available estimates for new retinitis (~0.01 per 100,000 person-years or <1,000 cases).

Applying the percentages of keratitis cases that result in mild or worse uniocular vision impairment^11,13,14^ to our best-estimates of the number of individuals with epithelial and stromal HSV keratitis globally in 2016, approximately 230,000 people worldwide could have newly experienced some degree of vision impairment caused by HSV keratitis in 2016, of which ~100,000 were caused by epithelial HSV keratitis and ~130,000 by stromal HSV keratitis.

## Discussion

We estimated that, in 2016, 1.7 million people had HSV keratitis, equivalent to an incidence of 24.0 per 100,000 person-years, the majority of whom had epithelial keratitis. Of these 1.7 million individuals, approximately 230,000 people (~15%) may have newly acquired some degree of uniocular vision impairment (mild or worse) associated with HSV keratitis in 2016. These figures likely underestimate the full burden of ocular disease due to HSV, as data were more limited for rarer but potentially more vision-impairing conditions such as HSV uveitis and retinitis. We tentatively estimated that around 0.1 million cases of HSV uveitis might have occurred in 2016, although the fractions of uveitis alone and episodes that were concurrent to other ocular HSV disease were unknown.

### Strengths and limitations

Our study represents the most recent review of the literature on HSV keratitis incidence and the only review to our knowledge of the incidence of HSV keratitis and all other clinically relevant ocular disease caused by HSV infections. We were able to generate the first global estimates of the number of people separately with epithelial and stromal HSV keratitis, and with new versus recurrent keratitis. We also carried out a quality assessment of studies identified to have incidence data to increase the reliability of our results.

However, our review and resultant estimates have several limitations. First, our literature search was devised by expert co-authors but, due to resource constraints, conducted by one reviewer and limited to two key databases identified as having appropriate levels of coverage.^42^ We identified relatively few available studies, and even fewer studies which met our quality criteria,^15,16,28,30,32,35,36^ limiting the extent to which we could speculate with any confidence on global disease burden. Population-based surveys of vision impairment and eye disease have provided a large number of epidemiological estimates for chronic conditions such as cataract,^43^ but fewer such studies are available for short-lasting, episodic, or less common conditions such as those associated with HSV. As there was a lack of good-quality data on the incidence of HSV uveitis and retinitis we may have failed to capture an important contributor to the magnitude of vision impairment due to ocular HSV. Also quality data were lacking for blepharo-conjunctivitis, which rarely affects vision but may cause ocular discomfort and morbidity. Furthermore, although we did estimate the global burden of HSV keratitis, our estimates are based on at most three of four studies,^28,30,32,36^ meaning there is still considerable uncertainty. Our quality assessment aimed to mitigate some of the potential issues around study biases, while the 95% CI around our pooled estimates were an attempt to reflect the within- and between-study variation in the contributing estimates for incidence.

Second, in the absence of laboratory testing/typing, there is a degree of uncertainty where diagnosis is solely based on clinical presentation,^9^ which is the case for most of the contributing studies. Misdiagnosis may lead to over- or underestimation of incidence. Disaggregation to ‘highly probable’ clinical certainty was done by one study only, for HSV keratitis.^36^ The discrepancy between the incidence of all suspected cases and the incidence of highly probable cases only in this study highlighted possible limitations of estimating HSV ocular disease incidence in the absence of routine confirmatory lab testing. Studies also had other methodological differences. For example, incidence could be measured based on the number of individuals or based on the number of episodes, where an individual may have two or more episodes of clinical disease recorded.

Third, differences may exist in disease incidence across populations, particularly by age and by geographic location. The paucity of estimates meant we were unable to carry out a stratified analysis of incidence, and our global burden estimates were derived by applying one single pooled estimate of incidence per clinical entity universally across world population estimates. Of particular note, HSV ocular disease incidence is likely to be closely related to rates of HSV-1 infection. All the studies contributing data to our pooled estimates were conducted either in Europe or in the USA. The WHO Americas and Europe regions have the lowest prevalence of oral HSV-1 infection of all WHO regions,^1^ meaning the incidence of HSV ocular disease is likely to be higher outside Europe and the Americas, and our figures for the global burden of disease are therefore likely to be underestimates. However, the relationship between HSV infection rates and ocular disease rates is unclear, as there may be contributing factors such as the age at which individuals generally acquire infection, the site of original HSV-1 infection (oral versus genital), and other factors.^44^

Fourth, the incidence of HSV ocular disease may have changed over time, and since most data came from older studies, this may have led to an under- or over-estimation of current disease rates. Time trends are difficult to detect with only a limited number of estimates, however, two studies reported on the incidence of any HSV ocular disease and any HSV keratitis in the same population, between 1950 and 1982^28^ and 1976–2007.^32^ An increase in incidence with time was observed in the older study,^28^ while the more recent study found stable incidence.^32^ However, excluding studies based on study year would have further reduced the already limited number of available estimates for pooling. Furthermore, older studies may actually better reflect settings outside of Europe and the USA than newer studies, as historic infection rates in Europe and the USA may be closer in line with current prevalence in other regions.^45^

Last, disease burden estimates based on overall incidence give information on the total number of affected individuals, which is useful for appreciating the extent to which HSV eye disease is a public health problem. However, they have limited utility for understanding how quality of life is affected. WHO’s 2019 *World Report on Vision* noted that understanding the burden of unilateral vision impairment is an important gap in the global epidemiology of eye health that requires further research.^46^ To partly address this, we tentatively applied published estimates of uniocular vision impairment to our pooled incidence estimates for HSV keratitis. The estimated proportions of cases with vision impairment <6/12 come from studies in Europe and USA where evidence-based treatment modalities for ocular HSV, including topical antiviral agents and in certain situations topical steroids,^14,47^ are likely to be more available than in low-income parts of the world. It is therefore likely that the estimates of vision impairment are an underestimate of the actual morbidity due to ocular HSV.

### Impact and conclusions

Around 1.7 million people may have experienced HSV keratitis in 2016. HSV uveitis may have contributed an additional 0.1 million cases in 2016 (depending on the extent to which it presented concurrent to other conditions), which would bring the total to over 1.8 million cases. Based on our incidence estimates, some 230,000 people with HSV keratitis may have newly acquired uniocular vision impairment <6/12 in 2016. Our burden estimates are likely to be underestimates, however, as they were based on studies in the USA and Europe, which have lower HSV-1 prevalence and wider availability of treatment than in many other settings. Improved access to evidence-based treatment in low- and middle-income countries would reduce ocular morbidity globally. New HSV prevention methods, such as vaccines, could also have a considerable public health impact in reducing ocular HSV disease incidence and associated morbidity including uniocular vision impairment, in addition to broader benefits for HSV-associated genital, oral, and neonatal disease.

## Supplementary Material

Supplemental Material
