# Supplementary material for: INCIDENCE OF HERPES SIMPLEX VIRUS KERATITIS AND OTHER OCULAR DISEASE: GLOBAL REVIEW AND ESTIMATES
Source: Ophthalmic Epidemiol. 2021 Oct 8;29(4):353–62. doi: 10.1080/09286586.2021.1962919 (PMC9397127; doi:10.1080/09286586.2021.1962919)
Supplement: Supplemental Material [file IOPE_A_1962919_SM0362.pdf]

Annex: Incidence of herpes simplex virus keratitis and other ocular disease: systematic review and global estimates

Mr Ian McCormick, MSc, Dr Charlotte James, PhD, Professor Nicky J. Welton, PhD, Professor Philippe Mayaud, Dr Katherine M. E. Turner, PhD, Dr Sami L. Gottlieb, MD, Professor Allen Foster, and Dr Katharine J. Looker, PhD

Further methods

Box A1. Medline search terms used in the literature search. Equivalent search terms used in Embase search.

1. exp keratitis, herpetic/ or exp keratitis, dendritic/
2. ((herpe* or simplex or hsv*) adj4 (blephar* or conjunctiv* or keratoconjunctiv* or cornea* or kerati* or dendr* or epithel* or endothel* or stroma* or disciform or uveiti* or panuveiti* or retiniti* or retinopath* or necroti* or arn or porn or nonnecroti* or ocular or eye*)).tw.
3. exp Uveitis/ or Iridocyclitis/ or exp Retinitis/ or Retinal Necrosis Syndrome, Acute/ or exp Keratitis/ or exp Keratoconjunctivitis/ or exp CONJUNCTIVITIS/ or exp BLEPHARITIS/
4. (herpe* or simplex or hsv*).tw.
5. 3 and 4
6. 1 or 2 or 5
7. Incidence/ or inciden*.tw.
8. Prevalence/ or prevalen*.tw.
9. epidemiolog*.tw.
10. natural history.tw.
11. longitudinal*.ab,ti.
12. prospective*.ab,ti.
13. cohort*.ab,ti.
14. (follow up or follow*up or follow* up).ab,ti
15. trial.ab,ti.
16. RCT*.ab,ti.
17. time to event.ab,ti.
18. burden.tw.
19. rate.tw.
20. (length or duration).tw.
21. episode.tw.
22. recur*.tw.
23. or/7-22
24. 6 and 23
25. exp animals/ not humans.sh.
26. 24 not 25
27. limit 26 to English language

Further results

Table A1. Incidence data identified in the literature search, by study (listed in order of earliest to latest data collection period).

Author name (Year published)	Study country (Study years)	Population; denominator (N)	Study design	Strengths	Limitations	Age of HSV cases (ave; range)	HSV clinical entity		No. of HSV cases	Incidence (95%CI) (cases per 100,000 person-years)	Meets quality criteria?
Liesegang (1989)[28]	USA (1950-1982)	All residents of Rochester, Olmstead County, MN ("predominantly white, middle-class"); N not reported	Retrospective review of medical records linkage system	Well-defined denominator population; case ascertainment from outpatient and inpatient records of all local care providers; 33 year review period; clear case definitions	No lab confirmation of HSV in most cases; reliance on accuracy of medical records; non-diverse study population	Mean 37.1 at first episode	Any HSV ocular disease	New	122	8.4 (6.9-9.9)	Y
								Recurrent	172[d]	12.3[f]	Y
								All	294[d]	20.7 (18.3-23.1)	Y
							Any keratitis	New	84[a]	6.2[c]	Y
								Recurrent	164[d]	12.0[c,f]	Y
								All	248[d]	18.2[c]	Y
							Epithelial keratitis	New	77[a]	5.6	Y
								Recurrent	135[d]	10.0[f]	Y
								All	212[d]	15.6	Y
							Stromal keratitis	New	7[a]	0.6	Y
								Recurrent	29[d]	2.0[f]	Y
								All	36[d]	2.6	Y
							Uveitis	New	5[a]	0.4	Y
								Recurrent	20[d]	1.5[f]	Y
								All	25[d]	1.9	Y
							Blepharo-conjunctivitis	New	66[a]	4.2	Y
								Recurrent	54[d]	3.5[f]	Y
								All	120[d]	7.7	Y
Norn (1970)[11]	Denmark (1958-1964)	Copenhagen; N = approx. 700,000	Retrospective review of single hospital case series	7 year review period	No lab confirmation of HSV; single hospital case ascertainment; limited description of methods and results	Unknown	Epithelial keratitis	All	157	5.9[e]	N
Whitcher (1976)[29]	Tunisia (1972-1973)	Patients attending l'Institut d'Ophtalmologie de Tunis (national eye hospital in Tunisia); N = "five million"	Prospective hospital-based case series	Prospective 1 year study period; clear case definitions	Virus isolation HSV confirmation only; case ascertainment acknowledged as insufficient for defined denominator population	Unknown	Epithelial keratitis	All	48	1.4	N
Mortensen (1979)[30]	Denmark (1976-1978)	Funen county; N = 446,223	Prospective multiple clinic-based case series	Prospective 2 year study period; well-defined denominator population; case ascertainment from community ophthalmologists	No lab confirmation of HSV; limited description of methods	Mean 46.5; range 4-84	Epithelial keratitis	All	107	12.0	Y
Ribaric (1976)[31]	Croatia	Rijeka region; N = 800,000	Retrospective review of	18 year review period	No lab confirmation of HSV; denominator	Unknown	Any keratitis	All	584	4.1	N

	(Not reported)		'ambulatory and clinical' cases		population not well-defined; case ascertainment not described; no case definition reported						
Young (2010)[32]	USA (1976-2007)	All residents of Olmstead County, MN; N not reported	Retrospective review of medical records linkage system	Well-defined denominator population; case ascertainment from outpatient and inpatient records of all local care providers; 32 year review period; clear case definitions; excluded uncertain cases	No lab confirmation of HSV in most cases; reliance on accuracy of medical records; non-diverse study population	Mean 43; range 1-91	Any HSV ocular disease	New	394	11.8 (10.6-13.0)	Y
							Any keratitis	New	297	9.2 (8.1-10.3)	Y
							Epithelial keratitis	New	233	7.0[h]	Y
							Stromal keratitis	New	64	1.9[h,i]	Y
							Uveitis	New	2	0.060[h]	Y
							Retinitis	New	0	0[h,l]	Y
							Blepharo-conjunctivitis	New	79	2.4[h]	Y
Paivonsalo-Hietanen (1997)[33]	Finland (1980-1982, 1988)	Out-patients and in-patients of Turku University Hospital Eye Clinic; hospital district for N = 457,842 (1980), N = 472,540 (1988)	Retrospective cohort, review of hospital records	Well-defined denominator population for referral centre; 3 year and 1 year review periods	No lab confirmation of HSV; no case definition reported	Unknown	Uveitis	New	6	0.3[j]	N
Tran (1994)[34]	Switzerland (1990-1993)	New uveitis patients at Lausanne University eye clinic; referral centre for N = 600,000	Prospective hospital-based case series	Prospective 3 year study; clear case definition	Serology HSV confirmation only; denominator population not clearly explained	Mean 43; range 6-92	Uveitis	New	21	0.8[j]	N
Stanzel (2014)[35]	USA (1998-1999)	Nine Northern Californian communities: Oakland, Richmond, Walnut Creek, South San Francisco, Redwood City, Hayward, Fremont, Milpitas; N not reported	Retrospective review of health program medical records	Well-defined denominator populations selected to be representative of Northern California; case ascertainment from outpatient and inpatient records of all local care providers; 1 year review period; clear case definitions; excluded uncertain cases	No lab confirmation of HSV in most cases; reliance on accuracy of medical records/ ICD-9 coding	Mean 51.4; range 1-93	Any HSV ocular disease	New<br>Recurrent<br>All	71<br>59<br>130	6.8 (5.3-8.6)<br>5.7 (4.3-7.3)<br>12.5[b]	Y<br>Y<br>Y
Muthiah (2007)[15]	UK (2001-2002)	National; N not reported	Prospective national surveillance reporting study	Prospective 1 year study; well-defined denominator population; clear case definition; PCR confirmation	Case ascertainment via surveillance reporting system and follow-up questionnaire	Mean 54.3; range 13-85	Retinitis	New	6	Range 0.010-0.012[j,m]	Y
Labetoulle (2005)[36]	France (2002)	National; N = 60,186,184	Prospective multicentre case series	Prospective study; case ascertainment across 412 ophthalmologists representative of gender, geography and practice type nationally; clear case definitions; incidence reported for all and highly probable cases separately	No lab confirmation of HSV; annual incidence extrapolated from a 3 month study period; participating ophthalmologists (412/1595 contacted) self-selected	For n=357: <20 = 4.5% 20-60 = 55.9% 60+ = 39.6%	Any keratitis	New<br>Recurrent<br>All<br>Highly probable	145<br>202<br>357[g]<br>292	13.2 (10.4-15.9)<br>18.3 (14.6-22.1)<br>31.5 (25.5-37.5)<br>25.8 (21.2-30.4)	Y<br>Y<br>Y<br>Y
							Epithelial keratitis	All	190	22.0 (18.7-25.4)	Y
							Stromal keratitis	All	81	9.2 (7.0-11.3)	Y

Cochrane (2012)[16]	UK (2007-2008)	National; N = 60,975,000	Prospective national surveillance reporting study	Prospective 1 year study; well-defined denominator population; clear case definition; PCR confirmation	Case ascertainment via surveillance reporting system and follow-up questionnaire	Range 10-94	Retinitis	New	9	0.013[j]	Y
Adhin (2012)[37]	Suriname (2008-2010)	Patients attending the ophthalmic department of Academic Hospital Paramaribo (only ophthalmic centre in Suriname); N = 524,143	Prospective hospital-based case series	Prospective study; attempted to confirm HSV diagnoses with PCR	Case ascertainment from single tertiary centre for national population; annual incidence calculated on first 2 months of the study only (n=37/91) as there was a drop in cases reported after this time; limited description of methods and results; incidence value determined including PCR-negative cases; no HSV detected in 50% cases clinically diagnosed as HSV keratitis	Median 41.5; range 0-88	Any HSV ocular disease	All	37	11.4 (4.8-18.1)	N
Gómez-Mariscal (2020)[38]	Spain (2014-2017)	Patients attending eye emergency service at Ramón y Cajal University Hospital, Eastern Madrid city & north eastern metropolitan area; N = 566,455	Retrospective review of single hospital records	Well-defined denominator population; 4 year review period	No lab confirmation of HSV; no case definition reported	Mean 54.8; standard deviation ±18.1 years	Uveitis	New<br>Recurrent<br>All	60<br>41[d]<br>118[d,k]	-<br>-<br>0.067	N<br>N<br>N

"Cases" refers to individuals except where stated. Incidence is annual incidence: incidences were adjusted to account for length of study. Some reported incidences do not exactly match incidence calculated from number of cases, denominator and length of study: reported incidence always used in preference over calculated incidence as it is assumed reported incidences are adjusted or otherwise corrected for some unknown factor. [a]Number of cases calculated from data reported in study. [b]Incidence estimate calculated by combining mutually exclusive incidences of new and recurrent disease. [c]Incidence estimate calculated by combining separate epithelial and stromal keratitis incidences to derive an any keratitis estimate. Assumption that any keratitis other than epithelial or stromal (e.g. disciform) is rare and contributes a negligible number of cases. [d]Incidence estimate based on number of episodes rather than individuals (i.e., individuals can be counted more than once). [e]Based on 287 episodes. [f]Incidence estimate calculated by subtracting incidence of new disease from incidence of all disease (new and recurrent combined). [g]10 cases missing data on classification of new or recurrent and 85 on keratitis form. [h]Estimated from no. of cases/no. of cases of any HSV ocular disease * incidence of any HSV ocular disease. This method will not give the exact incidence, as reported incidences are age- and sex-adjusted. Number of cases of any HSV ocular disease also includes 16 cases of conjunctivitis alone, for which we did not calculate an incidence estimate. [i]Calculated by assuming number of cases of stromal keratitis equals no. of cases of any keratitis – no. of cases of epithelial keratitis. Assumption that any keratitis other than epithelial or stromal (e.g. disciform) is rare and contributes a negligible number of cases. However, the paper does state that there were 64 patients who presented with "other forms of keratitis [other than epithelial keratitis]", which implies that these cases were not solely stromal keratitis cases. [j]Derived from (no. of HSV cases/no. of cases due to any cause) * incidence due to any cause. Two incidence estimates given in paper; midpoint used for pooling. [k]Includes 17 episodes unclassified as either new or recurrent. [l]Non-zero values used for pooling (0.0001 cases; incidence 0.00001 per 100,000 person-years). [m]Two incidence estimates given in paper; midpoint used for pooling.

Table A2. Bias/quality assessment (based on the Newcastle-Ottawa scale), by clinical entity.

Author name (Year published)	Study country (Study years)	HSV clinical entity		No. of HSV cases	Incidence (95%CI) (cases per 100,000 person-years)	Quality assessment criteria			
						Are cases well defined?	Is the study population (denominator) well defined?	Is the study designed to capture all cases within the study population?	Are the study cases and populations representative of a general population, without major selection, referral or design bias?
Liesegang (1989)[28]	USA (1950-1982)	Any HSV ocular disease	All	294	20.7 (18.3-23.1)	*	*	*	*
Stanzel (2014)[35]	USA (1998-1999)	Any HSV ocular disease	All	130	12.5	*	*	*	*
Adhin (2012)[37]	Suriname (2008-2010)	Any HSV ocular disease	All	37	11.4 (4.8-18.1)	*	*	-	-
Liesegang (1989)[28]	USA (1950-1982)	Any HSV ocular disease	New	122	8.4 (6.9-9.9)	*	*	*	*
Young (2010)[32]	USA (1976-2007)	Any HSV ocular disease	New	394	11.8 (10.6-13.0)	*	*	*	*
Stanzel (2014)[35]	USA (1998-1999)	Any HSV ocular disease	New	71	6.8 (5.3-8.6)	*	*	*	*
Liesegang (1989)[28]	USA (1950-1982)	Any HSV ocular disease	Recurrent	172	12.3	*	*	*	*
Stanzel (2014)[35]	USA (1998-1999)	Any HSV ocular disease	Recurrent	59	5.7 (4.3-7.3)	*	*	*	*
Liesegang (1989)[28]	USA (1950-1982)	Any keratitis	All	248	18.2	*	*	*	*
Ribaric (1976)[31]	Croatia (Not reported)	Any keratitis	All	584	4.1	-	-	-	-
Labetoulle (2005)[36]	France (2002)	Any keratitis	All	357	31.5 (25.5-37.5)	*	*	*	*
Liesegang (1989)[28]	USA (1950-1982)	Any keratitis	New	84	6.2	*	*	*	*
Young (2010)[32]	USA (1976-2007)	Any keratitis	New	297	9.2 (8.1-10.3)	*	*	*	*
Labetoulle (2005)[36]	France (2002)	Any keratitis	New	145	13.2 (10.4-15.9)	*	*	*	*
Liesegang (1989)[28]	USA (1950-1982)	Any keratitis	Recurrent	164	12.0	*	*	*	*
Labetoulle (2005)[36]	France (2002)	Any keratitis	Recurrent	202	18.3 (14.6-22.1)	*	*	*	*
Labetoulle (2005)[36]	France (2002)	Any keratitis	Highly probable	292	25.8 (21.2-30.4)	*	*	*	*
Liesegang (1989)[28]	USA (1950-1982)	Epithelial keratitis	All	212	15.6	*	*	*	*
Norn (1970)[11]	Denmark (1958-1964)	Epithelial keratitis	All	157	5.9	*	*	-	-
Whitcher (1976)[29]	Tunisia (1972-1973)	Epithelial keratitis	All	48	1.4	*	*	-	-
Mortensen (1979)[30]	Denmark (1976-1978)	Epithelial keratitis	All	107	12.0	*	*	*	*
Labetoulle (2005)[36]	France (2002)	Epithelial keratitis	All	190	22.0 (18.7-25.4)	*	*	*	*
Liesegang (1989)[28]	USA (1950-1982)	Epithelial keratitis	New	77	5.6	*	*	*	*

Liesegang (1989)[28]	USA (1950-1982)	Epithelial keratitis	Recurrent	135	10.0	*	*	*	*
Liesegang (1989)[28]	USA (1950-1982)	Stromal keratitis	All	36	2.6	*	*	*	*
Labetoulle (2005)[36]	France (2002)	Stromal keratitis	All	81	9.2 (7.0-11.3)	*	*	*	*
Liesegang (1989)[28]	USA (1950-1982)	Stromal keratitis	New	7	0.6	*	*	*	*
Liesegang (1989)[28]	USA (1950-1982)	Stromal keratitis	Recurrent	29	2.0	*	*	*	*
Liesegang (1989)[28]	USA (1950-1982)	Uveitis	All	25	1.9	*	*	*	*
Gómez-Mariscal (2020)[38]	Spain (2014-2017)	Uveitis	All	118	0.067	-	*	-	*
Liesegang (1989)[28]	USA (1950-1982)	Uveitis	New	5	0.4	*	*	*	*
Paivonsalo-Hietanen (1997)[33]	Finland (1980-1982, 1988)	Uveitis	New	6	0.3	-	*	*	*
Tran (1994)[34]	Switzerland (1990-1993)	Uveitis	New	21	0.8	*	-	-	-
Liesegang (1989)[28]	USA (1950-1982)	Uveitis	Recurrent	20	1.5	*	*	*	*
Muthiah (2007)[15]	UK (2001-2002)	Retinitis	New	6	Range 0.01-0.012	*	*	*	*
Cochrane (2012)[16]	UK (2007-2008)	Retinitis	New	9	0.013	*	*	*	*
Liesegang (1989)[28]	USA (1950-1982)	Blepharo-conjunctivitis	All	120	7.7	*	*	*	*
Liesegang (1989)[28]	USA (1950-1982)	Blepharo-conjunctivitis	New	66	4.2	*	*	*	*
Liesegang (1989)[28]	USA (1950-1982)	Blepharo-conjunctivitis	Recurrent	54	3.5	*	*	*	*

Studies not meeting the quality criteria are shown in grey. One estimate based on highly probable cases only also excluded from pooled estimates.

Table A3. Summary of uniocular vision impairment (World Health Organization threshold of mild or greater; any visual acuity <6/12) data identified in the literature search, by ocular HSV disease type and key study characteristics.

Author name (Year published)	Study country (Study years)	Population; N	Age of HSV cases (ave; range)	HSV clinical entity	Treatment	No. of HSV cases (eyes)	% <6/12	Length of follow-up
Liesegang (1989)[28]	USA (1950-1982)	All residents of Rochester, Olmstead County, MN ("predominantly white, middle-class"); N NR	Mean 37.1 at first episode	Any ocular HSV disease	Debridement, topical idoxuridine, vidarabine, trifluridine, topical steroids	131	22.1	Mean 9 years
Hodge (1997)[39]	USA (1984-1994)	HIV- individuals within catchment area of San Francisco General Hospital attending eye clinic for keratitis; N = 1800 and N = 48,200	Mean 35.4 (HIV-)	All keratitis	Combinations of topical trifluridine, topical steroids and oral acyclovir	27 (HIV-)	48.0	12 months
Claoue (1996)[40]	UK (1994)	Patients presenting to 3 eye clinics over 1 week at Moorfields Eye Hospital, London; N = 229	Range 21-90	Any ocular HSV disease	NR	42	40.0	Not clear
Bremner (1982)[24]	Australia (1964-1979)	Princess Margaret Hospital, Perth, W.A; children under 13 years; N NR	43.4% < 4 years; 56.6% 4-12 years	Epithelial keratitis	NR	145	18.6	NR
Norn (1970)[11]	Denmark (1958-1964)	Copenhagen; N = approx. 700,000	Unknown	Epithelial keratitis	NR	56	8.9	NR
Norn (1970)[11]	Denmark (1958-1964)	Copenhagen; N = approx. 700,000	Unknown	Stromal keratitis	NR	51	41.2	NR
Wilhelmus (1981)[12]	UK (1979)	Patients who previously presented to Moorfields Eye Hospital, London in 1974; N = 152	Unknown	Stromal keratitis[3]	NR	38	23.7[2]	5 years
Barron (1994)[13]	USA (1989-1992)	Patients at university eye clinics in San Francisco, New Orleans, Houston, Atlanta, Milwaukee, New York, Chicago, Philadelphia	Mean 46; range 15-82<br>Mean 44; range 17-83	Stromal keratitis	Topical prednisolone and trifluridine, oral acyclovir<br>Topical prednisolone and trifluridine	50<br>47	42.0<br>27.7	6 months
Wilhelmus (1994)[14]	USA (1989-1992)	Patients at university eye clinics in San Francisco, New Orleans, Houston, Atlanta, Milwaukee, New York, Chicago, Philadelphia	Mean 47; range 16-81[1]<br>Mean 49; range 13-79[1]	Stromal keratitis	Trifluridine and topical steroid<br>Trifluridine	52<br>42	32.7<br>33.3	6 months
Rodriguez (1995)[41]	USA (Not reported)	Patients at Massachusetts Eye & Ear Infirmary, Boston	Mean 52.9; range 19-78	Uveitis	Topical prednisolone, oral acyclovir (long-term)<br>Topical prednisolone, oral acyclovir (short-term)	13<br>7	46.2<br>85.7	Mean 5.9 years

NR: not reported. [1]Age data at randomisation, not follow-up. [2]Percentage calculated indirectly from information in text. [3]Not entirely clear how much vision impairment attributable to stromal HSV keratitis.

References

11. Norn, M.S., Dendritic (herpetic) keratitis. I. Incidence--seasonal variations--recurrence rate--visual impairment--therapy. *Acta Ophthalmol (Copenh)*. 1970; 48(1): 91-107.
12. Wilhelmus, K.R., Coster, D.J., and Donovan, H.C., Prognostic indicators of herpetic keratitis. Analysis of a five-year observation period after corneal ulceration. *Arch Ophthalmol*. 1981; 99(9): 1578-1582.
13. Barron, B.A., Gee, L., Hauck, W.W., et al., Herpetic Eye Disease Study. A controlled trial of oral acyclovir for herpes simplex stromal keratitis. *Ophthalmology*. 1994; 101(12): 1871-1882.
14. Wilhelmus, K.R., Gee, L., Hauck, W.W., et al., Herpetic Eye Disease Study. A controlled trial of topical corticosteroids for herpes simplex stromal keratitis. *Ophthalmology*. 1994; 101(12): 1883-1895.
15. Muthiah, M.N., Michaelides, M., Child, C.S., and Mitchell, S.M., Acute retinal necrosis: a national population-based study to assess the incidence, methods of diagnosis, treatment strategies and outcomes in the UK. *Br J Ophthalmol*. 2007; 91(11): 1452-1455.
16. Cochrane, T.F., Silvestri, G., McDowell, C., Foot, B., and McAvoy, C.E., Acute retinal necrosis in the United Kingdom: results of a prospective surveillance study. *Eye*. 2012; 26(3): 370-377.
24. Bremner, M.H., Ocular herpes simplex infections in children. *Aust J Ophthalmol*. 1982; 10(1): 27-29.
28. Liesegang, T.J., Melton, I.L.J., Daly, P.J., and Ilstrup, D.M., Epidemiology of ocular herpes simplex: Incidence in Rochester, Minn, 1950 through 1982. *Arch Ophthalmol*. 1989; 107(8): 1155-1159.
29. Whitcher, J.P., Dawson, C.R., Hoshiwara, I., et al., Herpes simplex keratitis in a developing country. Natural history and treatment of epithelial ulcers in Tunisia. *Arch Ophthalmol*. 1976; 94(4): 587-592.
30. Mortensen, K.K. and Sjolie, A.K., Keratitis dendritica. An epidemiological investigation. *Acta Ophthalmol (Copenh)*. 1979; 57(5): 750-754.
31. Ribaric, V., The incidence of herpetic keratitis among population. *Ophthalmologica*. 1976; 173(1): 19-22.
32. Young, R.C., Hodge, D.O., Liesegang, T.J., and Baratz, K.H., Incidence, recurrence, and outcomes of herpes simplex virus eye disease in Olmsted County, Minnesota, 1976-2007: the effect of oral antiviral prophylaxis. *Arch Ophthalmol*. 2010; 128(9): 1178-83.
33. Paivonsalo-Hietanen, T., Tuominen, J., Vaahtoranta-Lehtonen, H., and Saari, K.M., Incidence and prevalence of different uveitis entities in Finland. *Acta Ophthalmol Scand*. 1997; 75(1): 76-81.
34. Tran, V.T., Auer, C., Guex-Crosier, Y., Pittet, N., and Herbort, C.P., Epidemiological characteristics of uveitis in Switzerland. *Int Ophthalmol*. 1994; 18(5): 293-298.
35. Stanzel, T.P., Diaz, J.D., Mather, R., et al., The epidemiology of herpes simplex virus eye disease in Northern California. *Ophthalmic Epidemiol*. 2014; 21(6): 370-377.
36. Labetoulle, M., Auquier, P., Conrad, H., et al., Incidence of herpes simplex virus keratitis in France. *Ophthalmology*. 2005; 112(5): 888-895.
37. Adhin, M.R., Grunberg, M.G., Labadie-Bracho, M., and Pawiroredjo, J., Incidence of Alpha-Herpes virus induced ocular disease in Suriname. *J Med Virol*. 2012; 84(12): 1937-1942.
38. Gomez-Mariscal, M., De Arriba, F., Revenga, M., and Gonzalez-Lopez, J.J., Do Season and Environment Have a Role in the Incidence of Anterior Uveitis Attacks? *Ocul Immunol Inflamm*. 2020; 28(5): 786-790.
39. Hodge, W.G. and Margolis, T.P., Herpes simplex virus keratitis among patients who are positive or negative for human immunodeficiency virus: an epidemiologic study. *Ophthalmology*. 1997; 104(1): 120-124.

40. Claoue, C. and De Cock, R., The spectrum of herpes simplex virus disease of the anterior segment in the 1990s. *Acta Ophthalmol Scand*. 1996; 74(4): 407-410.
41. Rodriguez, A., Power, W.J., Neves, R.A., and Foster, C.S., Recurrence rate of herpetic uveitis in patients on long-term oral acyclovir. *Doc Ophthalmol*. 1995; 90(4): 331-340.
